# A review of the disruption of breastfeeding supports in response to the COVID-19 pandemic in five Western countries and applications for clinical practice

**DOI:** 10.1186/s13006-022-00478-5

**Published:** 2022-05-15

**Authors:** Sarah Turner, Bridget McGann, Meredith ’Merilee’ Brockway

**Affiliations:** 1 Manitoba Interdisciplinary Lactation Center (MILC), University of Manitoba, Winnipeg, Canada; 2 Children’s Hospital Research Institute of Manitoba, University of Manitoba, Winnipeg, Canada; 3 Department of Community Health Sciences, University of Manitoba, Winnipeg, Canada; 4 Department of Pediatrics and Child Health, University of Manitoba, Winnipeg, Canada

**Keywords:** Breastfeeding, COVID-19 pandemic, Virtual support, Professional support, Maternal experiences

## Abstract

**Background:**

The COVID-19 pandemic has significantly altered how breastfeeding support is provided, resulting in mixed breastfeeding outcomes and experiences for mothers. The World Health Organization has consistently supported breastfeeding from the beginning of the pandemic. However, recommendations from obstetrical and gynaecological societies within individual countries have varied in their alignment with this guidance, resulting in inconsistent recommendations. It is unknown how breastfeeding guidelines, maternal breastfeeding experiences, and breastfeeding initiation and duration compared across five Western countries. The current study is comprised of two parts, each with a different objective. Part One objective: to review pandemic-related changes in professional society guidelines on breastfeeding support in Australia, New Zealand, Canada, the United Kingdom, and the United States; and Part Two objective: to conduct a narrative review to summarize the evidence of how the pandemic has changed breastfeeding initiation, duration, and mothers’ breastfeeding experiences during the pandemic in these five countries and provide recommendations for clinical lactation support.

**Methods:**

We searched for indicators that are impactful on breastfeeding outcomes: skin-to-skin contact, rooming in, direct breastfeeding and breast washing, in the five countries mentioned above and compared these to the recommendations from the World Health Organization. Next, we conducted a narrative review of the literature from these five countries to explore how the pandemic altered breastfeeding outcomes and used this information to provide suggestions for clinical practice moving forward.

**Results:**

Recommendations on the four practices above differed by country and were not always in alignment with the World Health Organization recommendations. Mother-infant separation after birth in the United States was associated with a lower prevalence of breastfeeding initiation and duration. While some mothers reported positive breastfeeding experiences during the pandemic, many mothers indicated negative experiences related to decreased social and professional support.

**Conclusions:**

The pandemic can inform practice recommendations and can be viewed as an opportunity to permanently modify existing methods to support breastfeeding families. The use of virtual care increased during the pandemic and should continue with specific considerations for prioritizing in-person care. This will help to provide more timely and accessible support for breastfeeding mothers.

## Background

The COVID-19 pandemic has highlighted the challenges of promoting and supporting breastfeeding while managing the risk of an infectious and little-understood disease. From the beginning of the pandemic, the World Health Organization (WHO) has recommended mothers be encouraged and supported to breastfeed, regardless of COVID-19 infection status [1–3]. These recommendations include practicing rooming-in throughout the day and night, skin-to-skin contact and direct breastfeeding within one hour after birth. Early in the pandemic, the WHO assessed that the risks of not breastfeeding outweigh the risks of COVID-19 infection [1]. Despite these early and unequivocal recommendations that were reinforced throughout the pandemic, practices which were misaligned with the WHO recommendations and those which may compromise the establishment and maintenance of breastfeeding, have been widespread [4, 5].

Beginning in March of 2020, anecdotal reports of changes to maternal-infant hospital practices to help mitigate the spread of the SARS-CoV-2 virus began to emerge. Of particular concern were widespread separation of dyads upon birth, deterring direct breastfeeding, and in some cases discouragement of expressed milk feeding [6, 7]. Since breastfeeding is a proximity- and touch-dependent behavior, even seemingly minor changes in practices related to rooming in, such as recommending the use of an isolette or maintenance of two metres of distance from the infant, could compromise the establishment of breastfeeding [8]. An additional recommendation in some countries instructed mothers to wash their breasts before each feed. This recommendation was a notably novel phenomenon that emerged alongside the COVID-19 pandemic and was a point of concern for many clinicians, because it can hinder the establishment of breastfeeding. Breast washing before every feed can disrupt the neonate’s ability to find the nipple by scent [9], as well as being generally burdensome to a new dyad who are both learning how to breastfeed [10]. Further, this recommendation may also disrupt the maternal skin microbiome and alter establishment of the infant’s microbiome [11].

Preliminary evidence suggests a COVID-19 diagnosis is a risk factor for separation and early, unplanned, breastfeeding cessation, possibly due to changes in hospital breastfeeding practices [12]. Furthermore, an Italian study found lockdowns (i.e. stay at home orders, schools closures, and closures of workplaces) to be associated with decreased exclusive breastmilk feeding, even in cases of maternal COVID-19 negativity [13]. It is important to explore how recommendations to prevent the spread of COVID-19 through breastfeeding changed over time and consider the impact these recommendations may have had on breastfeeding outcomes during the pandemic.

### Context

This paper focuses on five countries (Australia, New Zealand, Canada, the United Kingdom, and the United States) that typically represent Western culture. While breastfeeding rates differ amongst these five countries, they are similar hegemonically in their Western medical belief systems. Additionally, these countries are all considered to be high income and have similar infant mortality rates (< 6/1000) [14]. As such, the potential impact of disruptions to breastfeeding on infant health is similar amongst these five countries [15]. In addition, these five countries often rely on the precautionary principle in their medical system delivery. The precautionary principle posits that if an action is suspected of causing significant harm, one should not wait until sufficient evidence of that harm is available to avoid the action [16]. The precautionary principle informed changes to many healthcare guidelines early in the COVID-19 pandemic, which may have affected hospital practices, maternal experiences, and breastfeeding initiation in these countries. Finally, the documents and literature published from these five countries were written in English, making them accessible to the authors.

### Objectives

This study has two parts. The objective of Part One is to review pandemic-related changes in professional obstetrical and gynaecological society guidelines on breastfeeding support in Australia, New Zealand (N.Z.), Canada, the United Kingdom (the U.K.), and the United States (the U.S.) from March to December 2020. The objective of Part Two is to summarize the existing evidence of how the pandemic has changed breastfeeding initiation, duration, and mothers’ experiences with breastfeeding support. A synthesis of Parts One and Two is presented as recommendations for future practice.

## Part One: Changes in professional guidelines during the pandemic

### Methods

Guidelines and recommendations published by obstetrical and gynaecological societies are used to inform many policies and procedures in the perinatal care setting. In the U.S. and Canada, practice in childbirth care is largely driven by the obstetrical profession, whereas in Australia, N.Z., and the U.K., obstetricians collaborate with midwives to drive practice trends. Globally, the WHO sets the standards of practice. In March of 2020, the WHO categorized various clinical interventions into three categories in their clinical management guidance in cases of suspected or confirmed COVID-19: “Do” (intervention is beneficial), “Don’t” (intervention is harmful) and “Consider” (conditional recommendation) [1]. To compare the WHO recommendations [1–3] to those of national recommendations, we obtained and reviewed the professional obstetrical and gynaecological society recommendations on breastfeeding in cases of suspected or confirmed maternal COVID-19 diagnosis in five Western countries: Australia, N.Z., Canada, the U.K., and the U.S. We searched for COVID-19 breastfeeding guidelines on the official website of each country’s professional obstetrical and gynaecological organizations. In cases where non-current guidelines were referenced on the website but no longer linked to, we utilized the Wayback Machine [17] to search for previous versions of the current documents. In cases where a document was known to have previous versions, but records were not found online, we requested them directly from representatives of the organization. Updates that could not be obtained by either of these methods were not included in the study. We focused on professional guidance released from March 2020 to December 2020 to align with the period in which the evidence on the risk of COVID-19 infection through breastfeeding changed the most rapidly. The relevant societies and documents are: The Royal Australian and New Zealand College of Obstetricians and Gynaecologists (RANZCOG) [18, 19], The Society of Obstetricians and Gynaecologists of Canada (SOGC) [20–24], the U.K.’s Royal College of Obstetricians and Gynaecologists (RCOG, who published guidance jointly with the Royal College of Midwives) [25–29], and the American College of Obstetricians and Gynecologists (ACOG) [30–33]. We focused on recommendations for four areas that were meant to mitigate the spread of COVID-19: skin-to-skin contact, rooming in during the hospital stay, direct breastfeeding (as opposed to feeding expressed milk), and breast washing. The WHO definitions [34, 35] of these four practices are in Table 1. The practices of skin-to-skin contact, rooming in and direct breastfeeding were of interest because they are evidence-informed recommendations that enhance breastfeeding outcomes, but which, by virtue of being sensory- and proximity-based, are also at risk of being compromised in the context of a pandemic [36, 37]. We also included breast washing before every feed in our analysis because it was a novel recommendation that emerged in response to the COVID-19 pandemic, and can be disruptive to the establishment of breastfeeding [10].

**Table 1:** WHO definitions of practices to support breastfeeding during the COVID-19 pandemic

Term	WHO Definition
Skin-to-skin	The practice where an infant is laid directly on the mother’s bare chest as soon as possible after birth.
Rooming In	The infant is either placed in a stand-alone cot by the bedside or is bed-sharing by attached side-car crib, in comparison to the infant being placed separate to the mother in a hospital nursery.^a^
Direct feeding	Infants who are fed directly at the breast.
Breast washing	Gently washing the breast with soap and warm water for at least 20 seconds prior to feeding if mother has just coughed on it.

^a^ In situations where two metre social distance from infant or use of an isolette or other barrier is recommended, the criteria for rooming in are not met

Using the WHO’s operational definitions of each practice (Table 1), we classified the professional guidelines on these four practices into three main categories, similar to the WHO classification [1]. When an organization explicitly or unequivocally recommended a practice, the item was categorized as “Yes.” When an organization recommended against a practice, it was categorized as “No.” For example, if an organization recommended “immediate isolation” of the infant, it was categorized as “No” for rooming in. In the case of breast washing, we added a category called “consider” to classify the Canadian recommendation that suspected or confirmed COVID-19 mothers consider cleansing their breast/chest before every feed. When a document did not mention a practice, it was classified as “Absent.”

### Results

**Fig. 1:**
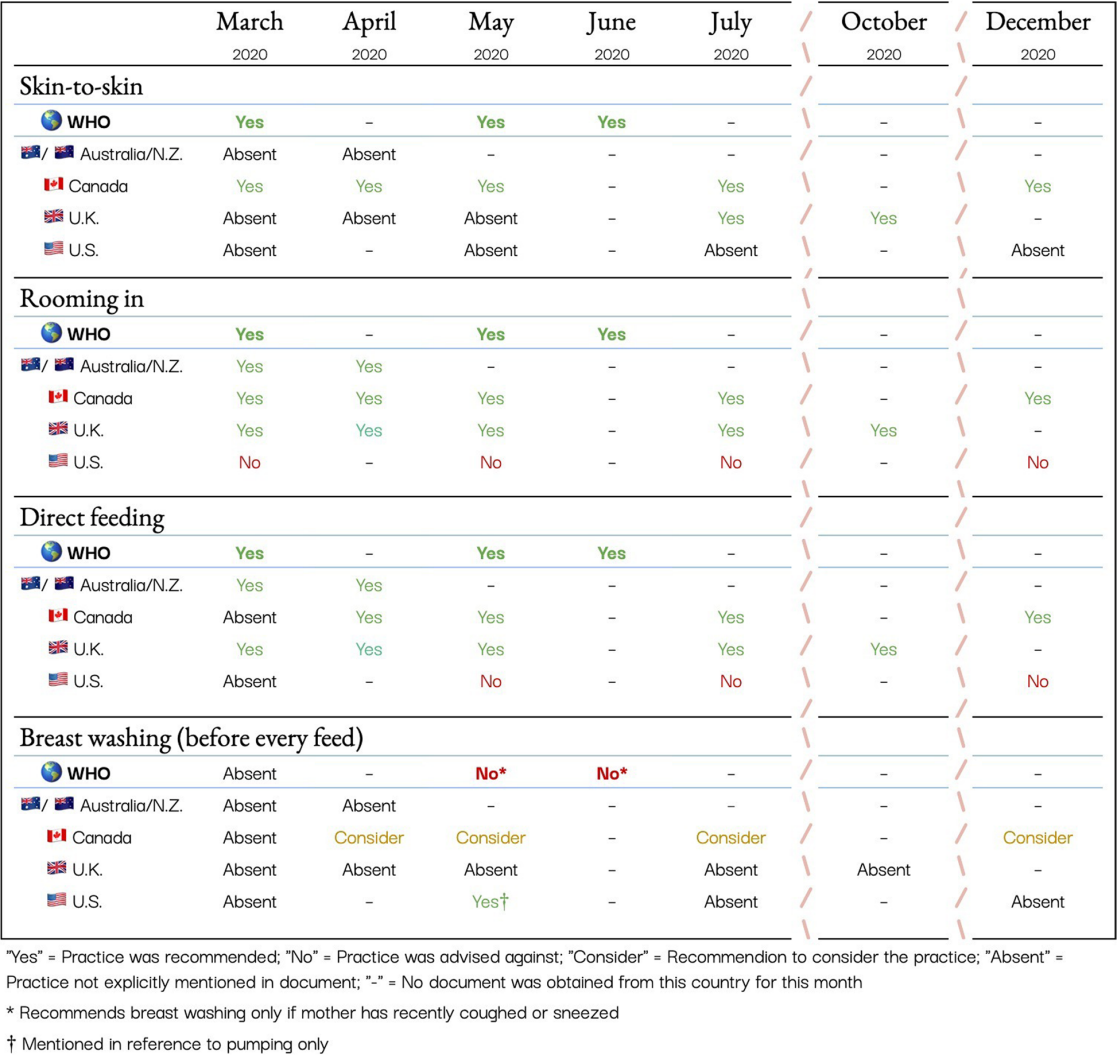
Analysis of Breastfeeding guidelines in Australia/New Zealand, Canada, the United Kingdom, and the United States in cases of confirmed or suspected maternal COVID-19 in 2020

Table 2 presents all of the obstetrical and gynaecological documents used in the current analysis. Consistently throughout the pandemic, the WHO recommended skin-to-skin contact, rooming in and direct breastfeeding (Fig. 1). A recommendation to wash breasts if the mother was sick and had coughed on them was instated in May 2020, however, it was explicitly stated that breast washing was otherwise not required. Individual country guidelines became more specific as evidence accumulated regarding the safety of breastfeeding and COVID-19.

**Table 2:**
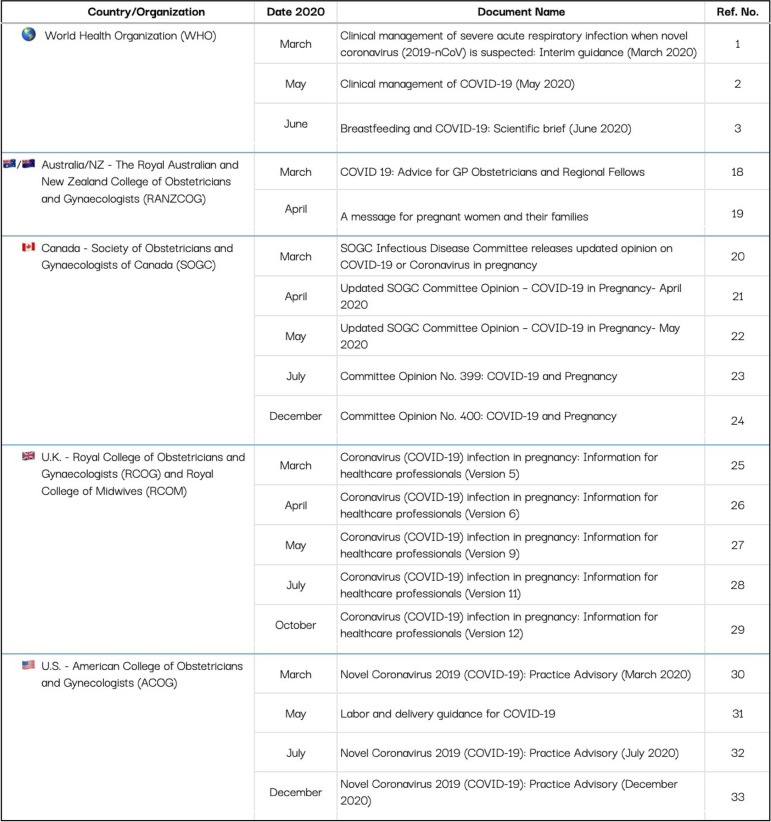
Analyzed obstetrical and gynaecological documents from Australia/New Zealand, Canada, the United Kingdom, and the United States

Canada was the only country to explicitly align with the WHO regarding skin-to-skin contact throughout the entire study period, while Australia/N.Z. and the U.K. were the only countries to consistently recommended direct breastfeeding. Australia/N.Z., Canada and the U.K. consistently recommended rooming in throughout the study period, aligning with the WHO recommendation. Conversely, the U.S. was inconsistent, ambiguous, or in explicit contradiction to WHO guidelines in their recommendations throughout the outlined time period. For example, in March, the U.S. recommended that an infant born to a mother with suspected or confirmed COVID-19 be treated as a PUI (Person Under Investigation) and isolated. The PUI language was removed from their documents in May, but they continued to advise that mothers be counseled on the use of an isolette or other barrier, or the maintenance of two meters of distance between the dyad, and advising the feeding of expressed milk over direct feeding.

While not included in Australia/N.Z. or U.K. guidelines, in April 2020, Canada began recommending that breast washing prior to a feed “could be considered”. In May 2020, the WHO clarified that to minimally disrupt breastfeeding, a mother should only wash the breast prior to a feed if she has recently coughed on it. However, the Canadian guidelines maintained the stance of considering breast washing before every feed throughout 2020. The U.S. recommended breast washing before pumping in May 2020, but did not address whether it should be done prior to direct feeding and did not advise on this practice at any other point during the pandemic.

### Discussion

During the period of March to December 2020, professional society guidelines in five Western countries varied, with some more consistently aligning with the WHO recommendations than others. The results of this study are consistent with a global survey of 33 countries, including four of the five we examined, that found misalignment of country-specific guidelines with the WHO recommendations [7]. In this study, none of the professional guidelines aligned perfectly with the practices advised by the WHO. With the exception of breast washing in Canada, Australia/N.Z., Canada, and the U.K. quickly moved to align with the WHO in response to growing confidence with the available evidence. However, the U.S. did not progress to align with the WHO recommendations throughout 2020 (Fig. 1). The U.S. was the only country that ever explicitly advised against any of the WHO recommendations, including recommending against rooming in. This indicates that the precautionary principle may have informed the U.S. guidelines throughout the pandemic, but suggests that the Australia/N.Z., the U.K., or Canada (with the exception of breast washing) more readily adapted to emerging evidence on the risks and benefits of breastfeeding during SARS-CoV-2 infection.

The misalignment of the U.S. guidelines with the WHO during 2020 not only have direct impact on U.S. populations, but also inform decisions made in other countries about breastfeeding during COVID-19. In an analysis of breastfeeding and newborn care guideline documents from low-, middle- and high-income countries, the Centers for Disease Control and ACOG statements were referenced in 13 of the 32 documents [7]. This indicates that the U.S. guidance was used to inform breastfeeding care guidelines and resulting practices in other countries. This is especially concerning for low- and middle-income countries where the additional infant deaths due to mother-infant separation and not breastfeeding are approximately 67 times greater than the additional infant deaths from COVID-19 infection [38].

Confusion around how to balance breastfeeding support and infant health in the context of a poorly understood pathogen is not a new phenomenon to breastfeeding care. It is known that early in the AIDS crisis, the introduction of infant formula was driven by the precautionary principle and contributed to early weaning and threatened breastfeeding [39]. Early inferences made about SARS-CoV-2 fueled concern that it might be spread from mother to infant via breastmilk or respiratory droplets. However, it is now well established that breast milk is protective against COVID-19 by supplying IgA and IgG antibodies to the infant that can bind to the SARS-CoV-2 spike protein [40, 41]. By considering parallels to the AIDS crisis, healthcare systems can more swiftly and precisely mitigate harm moving forward, and balance infant feeding-related risks in future pandemics.

While we are not able to directly assess the implementation of the aforementioned guidelines in hospital practice, changes to hospital practices in the summer of 2020 are reflected in Perrine et al. (2020). The authors examined changes in maternal-infant care in 1344 U.S. hospitals between July and August of 2020 [5]. Compared to pre-pandemic rates, 17.9% of hospitals had reduced in-person lactation support, and 72.9% of hospitals were discharging mothers less than 48 hours postpartum. Where mothers had confirmed or suspected COVID-19, 14% of hospitals discouraged skin-to-skin, 37.8% discouraged rooming in, 20.1% discouraged direct breastfeeding (recommending feeding expressed milk instead), and 28.6% separated dyads until COVID status was confirmed [5].

In the context of changing information in the weeks and months following the emergence of a pandemic, it is important that professional breastfeeding recommendations be clear, up-to-date, and reflect the best evidence in order to protect breastfeeding outcomes and postnatal experiences for families. In the U.S., the trend toward a precautionary approach to supporting breastfeeding lead to breastfeeding being de-prioritized. Critically evaluating how the pandemic affected the development of professional guidelines, and evaluating how the recommendations changed in response to a changing knowledge base, may help protect breastfeeding during future pandemics. Examining professional attitudes and logistical barriers to a rapid pandemic response, as well as cultural and structural barriers to implementation in a clinical context, may also be of benefit. A proactive rather than reactive response may reduce confusion, inconsistent care, maternal stress, and positively affect breastfeeding outcomes. We now turn our attention towards reviewing how changes to breastfeeding support, positive COVID-19 status, and isolation have impacted breastfeeding initiation and duration as well as mother’s experiences with breastfeeding.

## Part Two: Impact of COVID-19 on breastfeeding initiation, duration, and mother’s experiences of breastfeeding

### Methods

We conducted a narrative review of publications exploring how the pandemic restrictions, and positive COVID-19 status of the mother, impacted breastfeeding initiation, duration, and mothers’ self-reported breastfeeding experiences in Australia, N.Z., Canada, the U.K., and the U.S. Four literature searches were completed on PubMed using the key words, “breastfeed*”, “pandemic OR COVID-19” and either “Australia OR New Zealand”, “Canada”, “United Kingdom”, or “United States”. Results were limited to publications in 2020 and 2021. Inclusion criteria were as follows: original research study (i.e., not a commentary or review paper), participant population living in Australia, N.Z., Canada, the U.K., or the U.S., and included the outcome of breastfeeding initiation, duration, or mothers’ experiences of breastfeeding during the pandemic. The Australian and N.Z. search returned 28 results, of which two met inclusion criteria [42, 43], the U.K. search returned 26 articles, of which two met inclusion criteria [44, 45], the U.S. search returned 63 articles, of which six met inclusion criteria [5, 46–50], and the Canadian search returned 29 articles, of which two met inclusion criteria [51, 52]. Two additional articles on breastfeeding initiation and duration in the U.S. were identified through searching the references of the identified publications and through RSS alerts of newly published articles of breastfeeding during the pandemic [12, 53].

### Results

Seven studies from the U.S. and one study from the U.K. showed that the COVID-19 pandemic was related to changes in breastfeeding initiation and duration [5, 45–47, 49, 50, 53]. No Australian, N.Z. or Canadian data on breastfeeding initiation or duration during the pandemic was found. In studies including COVID-positive mothers (all from U.S. samples) a reduction in breastfeeding initiation and duration was observed. Similarly, in a low-income U.S. sample not limited to COVID-positive mothers, breastfeeding exclusivity and duration declined during the pandemic. However, breastfeeding rates remained comparable to pre-pandemic rates in a general U.K. sample.

Among the studies including COVID-positive mothers, mother-infant separation emerged as the main barrier to establishing breastfeeding. In a U.S. study of 85 COVID-positive mothers in New York City, 58% were separated from their infants after birth [12]. None (0%) of the mothers who were separated from their infant were able to initiate breastfeeding in hospital, and only 12% were able to breastfeed when they arrived home. This is in contrast to 22% of non-separated mothers initiating breastfeeding in hospital, and 28% being able to breastfeed after arriving home. Among those who were separated from their infant at birth, 35% of mothers reported changing their feeding plan because of their illness, specifically due to separation and difficulty with latch [12]. Similar results were reported in another U.S. study that reviewed the neonatal and maternal outcomes of 70 COVID- positive mothers from 16 U.S. hospitals [50]. In this population, 51% of the dyads were separated after birth. Among those separated, 84% were feeding infant formula or donor milk exclusively, with only 14% feeding either exclusive expressed mother’s own milk, or expressed mother’s own milk with supplementation. None (0%) of these mothers were feeding directly at the breast. Among those who were allowed to room in, 85% were able to initiate direct breastfeeding (with or without supplementation), with only 18% providing infant formula or donor milk exclusively [50]. Similar trends were also observed in a study examining breastfeeding from one New York City hospital [47]. The authors compared the prevalence of separation and breastfeeding initiation between COVID-positive (*n* = 15) and COVID negative mothers (*n* = 64). All of the COVID-positive mothers were separated from their infant at birth, with only 33% direct breastfeeding to some degree. Among COVID-negative patients, 23% were separated from their infant, with 67% direct breastfeeding [47]. In contrast to the high separation rates stated above, a different U.S. sample of 82 neonates born to COVID-positive mothers from four hospitals in New York City, reported 83% of infants were allowed to room in with their mothers [46]. However, infants were kept in a closed isolette, which does not meet the WHO definition of rooming in [34]. At 5–7 days post-partum, 78% of the 82 infants were receiving at least some breastmilk.

In studies not limited to COVID-positive mothers, the prevalence of breastfeeding initiation and duration remained mostly stable aside from one study including a low-income sample from the U.S. [53]. This study included a sample of 2426 low-income mothers who participated in the Special Supplemental Nutrition Program for Women, Infants, and Children (WIC) in Southern California. The authors found that the prevalence of any breastfeeding at 6 months decreased from 48.7% before March 2020 to 38.6% after March 2020 [53]. Alternatively, in a large study of 1344 hospitals from across the U.S., 68% of hospitals reported that their exclusive breastfeeding rates during hospitalization stayed the same, while 11% reported an increase and 12% reported a decrease [5]. Using an online survey, Burgess et al. recruited 258 pregnant people from the U.S. and asked them if the COVID- 19 pandemic changed their infant feeding plans [49]. Only 3.1% indicated their infant feeding plan had changed, with 83% indicating they were now planning to breastfeed to provide better immune protection and due to fears of formula shortage. A single U.K. study of 316 mothers, reported that 72% of mothers who gave birth during the COVID-19 lockdown initiated breastfeeding; a proportion that was comparable to mothers who gave birth before the lockdown (76%) [45].

While quantitative evidence from the U.S. indicates that the pandemic has altered the prevalence of breastfeeding initiation and duration, especially for dyads who were separated at birth, it is important to also consider mother’s experiences of breastfeeding during this time. Our search strategy returned two Australian and/or N.Z. studies [42, 43], two studies from the U.K. [44, 45], one study from the U.S. [48], and two Canadian studies [51, 52] that examined maternal experiences of breastfeeding during the pandemic.

In one Canadian province, a sample of 335 mothers with infants less than 6 months of age completed an online survey with open and close-ended questions [52]. Mothers in this sample reported negative experiences related to the pandemic such as no social support, difficulty receiving professional help with breastfeeding techniques over the phone, trouble accessing specialized infant formulas, and fear of developing low milk supply due to the stress of living in the pandemic. These experiences are similar to another Canadian study that analyzed interviews with 57 post-partum mothers [51]. Mothers in this sample reported challenges with the lack of care provided in the hospital, absence of social support, and poor mental health, leading some to stop breastfeeding sooner than desired.

These themes are reflected in studies from the U.S., the U.K., Australia and N.Z. as well. In the U.S., mothers reported similar challenges of inadequacy of online breastfeeding support, as well as less social support, feelings of isolation, and challenges caring for an infant and older child at home at the same time [48]. In addition, U.S. mothers reported concerns about returning to the workplace while expressing breastmilk and finding a safe, non-contaminated place to do so. Two large online samples from the U.K. also found that many mothers struggled with having enough support to continue breastfeeding [44, 45]. An online survey of 316 mothers who gave birth during the lockdown found that 45% felt they were not receiving enough support with feeding [45]. A separate online survey of 1219 breastfeeding mothers, found that 27% experienced barriers with infant feeding such as lack of support, leading some to stop breastfeeding before they were ready [44]. These mothers also noted concerns with trying to juggle caring for older children without family support, lack of experience with breastfeeding in public and stress of pumping at a busy workplace for those who worked as health professionals. Finally, in Australia, a large online survey returned responses from 3364 women who were pregnant or had given birth since March 2020 [42]. Mothers reported concerns about lack of social support, having to ‘do it all alone’, and being deprived of antenatal classes, leaving them without the necessary information to feel prepared for the transition to parenthood. A sample of 364 mothers from Australia and N.Z. who were all breastfeeding at the time of data collection, reported concerns about lack of social interaction for their child, concerns about returning to work, and not being able to see family members [43].

Mothers from all five countries also reported positive experiences with breastfeeding during the pandemic lockdown, however, these were mentioned less frequently than negative experiences [42–44, 48, 51, 52]. Commonly cited positive factors included more time to focus on breastfeeding, bonding with baby without distractions, fewer visitors, greater partner support and delay of return back to work. These ‘silver linings’, provided some mothers the uninterrupted time they needed to establish breastfeeding without pressure to host visitors or busy schedules. However, two studies further explored subgroups of mothers with positive experiences, and found that these were mostly reported among mothers with higher income, fewer pre-existing mental health challenges, and less complicated births [44, 51].

## Moving beyond the pandemic: Recommendations for future practice

The pandemic can be viewed as a source of disruptive innovation and an opportunity to modify our existing methods to support breastfeeding families [54]. Healthcare is traditionally slow to adopt innovation, new policies, and procedures [55]. However, the dramatic and rapid changes to health care provision during the pandemic have rapidly forced changes to practice. Throughout the pandemic, the WHO maintained consistent recommendations to support breastfeeding; however, many mothers reported feeling that they did not receive adequate support [42, 44, 45, 51, 52]. During this time, most breastfeeding peer support groups and care from trained breastfeeding professionals transitioned to online platforms [56, 57]. While most mothers preferred in-person support, some enjoyed the convenience of receiving virtual care in the comfort of their own home [44]. In one study, most mothers who received virtual support found it helpful, with one-on-one video calls (86%), and social media (84%) being the preferred platforms of support [58]. As such, it is important to analyze which elements of online support should continue to be offered and consider which circumstances in-person support should be prioritized to incorporate into future breastfeeding support.

**Fig. 2:**
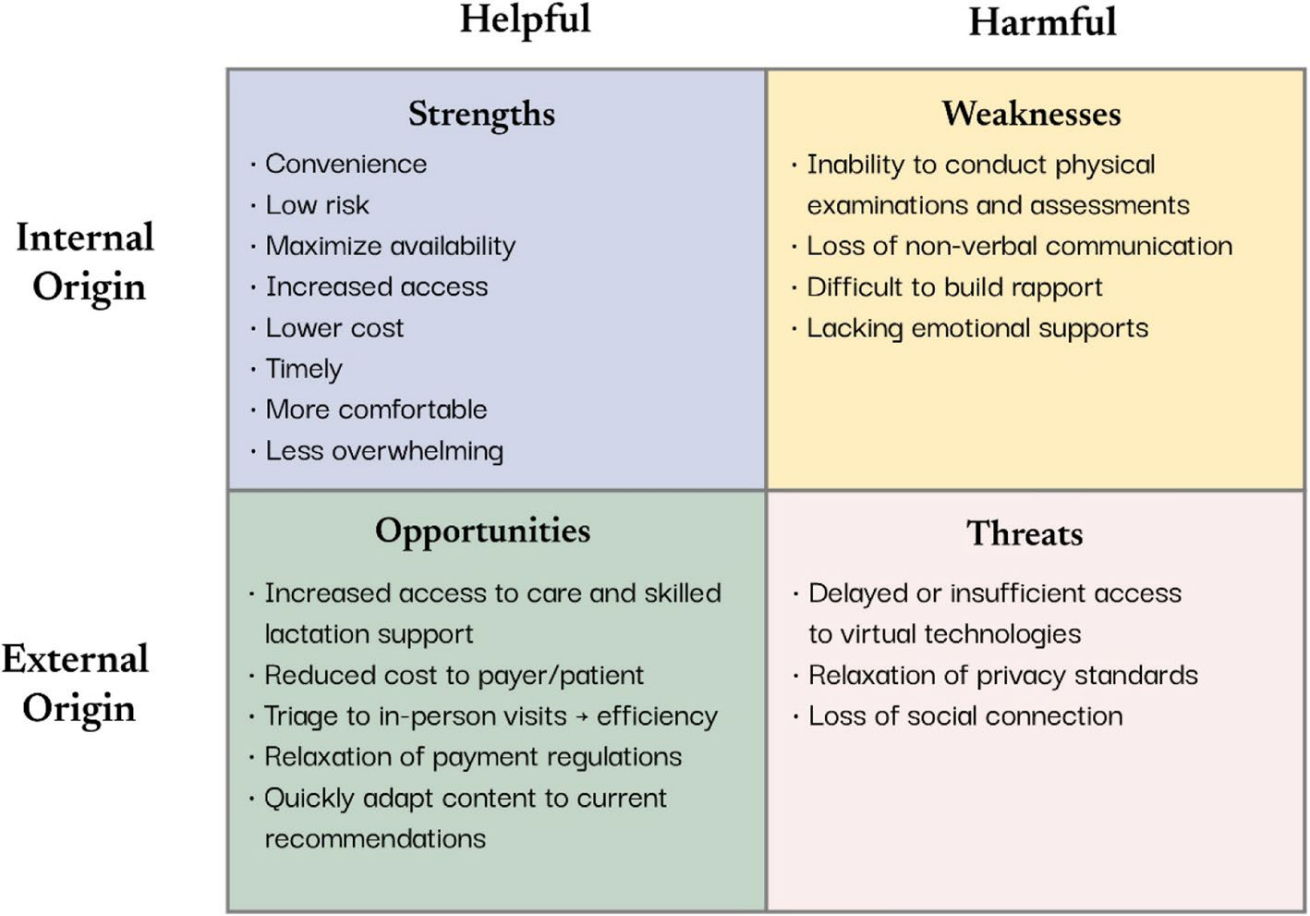
SWOT analysis (strengths, weaknesses, opportunities, and threats) of virtual breastfeeding care emerging from the pandemic

Through the lens of healthcare professionals who are providing breastfeeding support, we compiled the strengths, weaknesses, opportunities, threats (SWOT) of virtual care to consider which aspects may serve to augment existing breastfeeding support strategies. SWOT analyses [59] are common strategies used in quality appraisal and strategic planning to identify barriers and facilitators that can help to aid decision making [60]. Our SWOT consisted of a structured assessment that identified internal and external factors that could impact the implementation of virtual breastfeeding supports. Internal factors are evaluated as strengths and weaknesses imposed by the organization, which, in this case, is the healthcare provider’s workplace. External factors are evaluated as opportunities and threats imposed by societal influences such as demographics, socio-cultural belief systems, and economics. In the context of healthcare provision during the pandemic, this SWOT will allow practitioners to consider potential barriers and facilitators as they move forward in their practice using virtual support strategies to provide care (Fig. 2).

There are many *strengths* and *opportunities* to continuing to provide a virtual option for breastfeeding support. Increased convenience and ability to access these supports is an important consideration that can enhance accessibility to services. While not all mothers will have reliable internet access or connectivity (external - threat), providing virtual support can increase access to mothers who live in rural or remote settings [61]. Additionally, even in urban settings, virtual care reduces the need for the care provider or the mother to commute to physical locations during the postpartum period [62]. This reduction in commuting time increases the availability of the care provider and may help to reduce wait times for mothers to access care. Additionally, decreased commuting time as well as the reduced need for large spaces to accommodate groups, provides the opportunity to reduce costs to the healthcare system and payer [61]. Healthcare providers can also use virtual care to triage mothers based on their breastfeeding support needs. Triaging will allow mothers to be seen quicker and help to increase the efficiency of care provision.

While providing virtual care is an excellent augmentation to traditional breastfeeding support [63], there are some *weaknesses* and *threats* that should be considered. Despite over 87% of mothers in Australia, N.Z, Canada, the U.K, and the U.S., having access to the internet [64], this is not universal. Racialized populations, Indigenous communities, those living in rural communities, and those in lower income categories are significantly less likely to have internet access in their homes [65, 66]. This lack of access poses a threat and may further perpetuate existing healthcare inequities in access to reliable healthcare and breastfeeding support [67]. While calls-to-action to improve internet access increased over the course of the pandemic [68], meaningful initiatives to address these gaps are still lacking. Ensuring privacy standards and patient confidentiality in the online environment also poses a threat to virtual care. The pandemic has resulted in new opportunities for payment and regulatory standards for clinicians in conducting online breastfeeding support. However, providers need to be cognizant of privacy standards and perhaps implement additional security measures to ensure patient information remains confidential in the online environment [61].

In practical terms, the biggest weakness in providing virtual breastfeeding support is the inability to conduct physical assessments [61, 69]. Conditions such as tongue ties, as well as breast and suck assessments often require the practitioner to physically touch the mother and/or the infant [70]. As such, interactions that require a physical assessment should be prioritized to in-person care. As well, the pandemic restrictions necessitated the creation of innovative healthcare resources for practitioners to share with patients. Use of online resources such as teaching videos [71], information sheets [72], and online support groups [73] have expanded the breadth of resources available to practitioners and have increased accessibility to breastfeeding support. Finally, building rapport and using non-verbal communication strategies are limited in an online environment. These limitations may pose a threat to perceived social connection [56]. Developing strategies to enhance online presence or *webside manner*, such as proper positioning on-screen, creating a warm and professional environment, and maintaining eye-contact throughout the visit [74] may help to mitigate some of these threats.

While conducting care in the virtual environment may not be the ideal or preferred experience for all care providers and families, it does present several opportunities to streamline breastfeeding support to provide more efficient and equitable access [63]. Clinically, it is important for care providers to embrace the disruptive innovation of the pandemic and consider how they can integrate virtual options for breastfeeding support into their repertoire to enhance the care they provide. On a policy level, improving access to the internet for rural and marginalized communities may help mitigate health disparities that affect breastfeeding outcomes for vulnerable populations [63].

## Discussion

This paper aimed to 1) review the obstetrical and gynecological recommendations, from March to December 2020, of practices that impact breastfeeding in Australia, N.Z., Canada the U.K., and the U.S., and 2) review the available literature on how the pandemic-related changes to breastfeeding support altered breastfeeding initiation, duration and mothers’ experiences of breastfeeding in these countries. We also provide a discussion of the strengths and weaknesses of integrating virtual breastfeeding support into clinical practice.

Overall, we found that professional recommendations varied among countries, with the U.S. diverging the most from the WHO standards. Changes in hospital practices, particularly separating mother and infant after birth in U.S. hospitals, resulted in lower initiation and continuation of breastfeeding, especially among COVID-positive mothers. The consequences of mother-infant separation have been thoroughly outlined elsewhere, and the potential decreased risk of mother-to-child transmission of SARS-CoV-2 must be weighed against the risks to breastfeeding initiation, maternal mental health and mother-infant bonding [75].

Regardless of infection status, lockdowns and public health precautions implemented to mitigate the spread of COVID-19 resulted in many negative consequences for breastfeeding mothers, notably, lack of breastfeeding support. We view the pandemic as an opportunity to strengthen virtual breastfeeding supports and augment in-person care with virtual supports to reach more mothers.

The findings of this study must be considered through an equity lens. Previous research from New York City, has shown that Black and Latina women who gave birth during the pandemic had higher perceived healthcare discrimination than those who identify as White and were less likely to be breastfeeding upon hospital discharge [76]. In the U.K., Black and Minority Ethnic women were less likely to report a positive experience during the pandemic, compared to White women [44]. Finally, in Canada, mothers who were breastfeeding during the pandemic were significantly more likely to be living with a partner, have a graduate or professional degree and have a household income over $30,000, compared to those who were feeding infant formula or mixed feeding [52]. To provide appropriate breastfeeding supports for mothers during the pandemic, and into a post-pandemic world, the effects of the pandemic lockdowns, and changes in hospital practices, should be considered in the context of pre-existing social inequities.

### Limitations

This research has several limitations. In Part One, professional organizations often released guideline updates during the study period of March to December 2020, but retrospective versions of these documents were not always publicly available or obtainable from the organizations of interest. It is therefore possible that more versions were published during the period of March to December 2020 than are represented in these data. Secondly, organizations did not always provide clear operational definitions for each guideline, such as how they defined “rooming in” (given that use of an isolette or two metre of distance from infant would not be consistent with the WHO’s definition of breastfeeding), or whether “separation” of the maternal-infant dyad was referring to the period directly after birth or during the entire hospital stay. Thirdly, we do not have data on how closely the guidelines were followed in hospitals. It is likely that adherence to these guidelines varied substantially based on individual hospital policy. In the U.S. there is some published research on the implementation of maternal-infant separation policies in different hospitals, however, this does not exist on a national level for any of the other countries studied.

In Part Two, breastfeeding experiences were reported from self-selected samples and typically recruited through online modalities. Therefore, these results are subject to selection bias, and it is possible they do not reflect the experiences of the general population and those who are underrepresented in these samples. Additionally, while we structured our analysis around five countries with similar historical influences and belief systems around breastfeeding, these countries still differ in their breastfeeding rates and the pandemic may have had varying levels of impact on maternal experiences in each country. As well, recommendations for practice were applied to a generic Western context and will need to be tailored to individual countries based on available resources and healthcare delivery systems.

## Conclusions and future directions

Changes in hospital practices and community breastfeeding support due to the pandemic have resulted in sub-optimal breastfeeding initiation and duration, as well as challenging breastfeeding experiences for mothers. Preparedness is key in ensuring the most rapid response to a global pandemic. However, maternal and child health is often sidelined in the response to a crisis, even in the planning stages [77]. To rapidly respond to the emergence of a novel pathogen, researchers have called for a funded task force focused on human milk and infant feeding (Expert Panel Discussion at The Origins and Benefits of Biologically Active Components in Human Milk Conference, June 2021). This will help to ensure that when the next pandemic occurs, the infrastructure and networks are in place to develop our understanding of the new pathogen’s interactions with human milk soon as possible. The social complexity of breastfeeding and human lactation also requires a comprehensive approach that integrates multiple facets of society to ensure support is robust and sustainable enough to withstand the pressure introduced by a pandemic. Further, professional organizations should consider building decision-making tools and rapid response systems for producing and updating clear, comprehensive, and scientifically driven breastfeeding guidelines in an ongoing crisis. Collecting real-time data on hospital policies and breastfeeding support practices can help draw a clearer picture of how quickly professional recommendations are implemented in response to changes in the knowledge base and how these changes affect breastfeeding dyads. Finally, identifying the opportunities and strengths resulting from the pandemic can inform ongoing practice and ultimately protect vulnerable populations in a post-pandemic world. Increasing access to virtual breastfeeding support may result in reduced practical burdens on postpartum mothers, more efficient triage, and decreased cost of care.

## Data Availability

Not applicable.
